# Ferroptosis and Iron Homeostasis: Molecular Mechanisms and Neurodegenerative Disease Implications

**DOI:** 10.3390/antiox14050527

**Published:** 2025-04-28

**Authors:** Nurzhan Abdukarimov, Kamilya Kokabi, Jeannette Kunz

**Affiliations:** Department of Biomedical Sciences, School of Medicine, Nazarbayev University, Astana 010000, Kazakhstan; nurzhan.abdukarimov@nu.edu.kz (N.A.); kamilya.kokabi@nu.edu.kz (K.K.)

**Keywords:** ferroptosis, iron homeostasis, cell death, Alzheimer’s disease, Parkinson’s disease

## Abstract

Iron dysregulation has emerged as a pivotal factor in neurodegenerative pathologies, especially through its capacity to promote ferroptosis, a unique form of regulated cell death driven by iron-catalyzed lipid peroxidation. This review synthesizes current evidence on the molecular underpinnings of ferroptosis, focusing on how disruptions in iron homeostasis interact with key antioxidant defenses, such as the system Xc^−^-glutathione-GPX4 axis, to tip neurons toward lethal oxidative damage. Building on these mechanistic foundations, we explore how ferroptosis intersects with hallmark pathologies in Alzheimer’s disease (AD) and Parkinson’s disease (PD) and examine how iron accumulation in vulnerable brain regions may fuel disease-specific protein aggregation and neurodegeneration. We further surveyed the distinct components of ferroptosis, highlighting the role of lipid peroxidation enzymes, mitochondrial dysfunction, and recently discovered parallel pathways that either exacerbate or mitigate neuronal death. Finally, we discuss how these insights open new avenues for neuroprotective strategies, including iron chelation and lipid peroxidation inhibitors. By highlighting open questions, this review seeks to clarify the current state of knowledge and proposes directions to harness ferroptosis modulation for disease intervention.

## 1. Introduction

Cell death is a fundamental biological process with both physiological and pathological implications. While accidental cell death (ACD) occurs under extreme conditions such as severe mechanical stress or toxic chemical exposure, regulated cell death (RCD) follows orchestrated molecular pathways [1]. Among the various forms of RCD, apoptosis is the most extensively characterized [2]. Apoptosis plays a central role in embryonic development, tissue remodeling, immune regulation, and aging. It is a programmed mechanism designed for the controlled removal of unwanted, damaged, or potentially harmful cells. Apoptosis is characterized by distinctive morphological features, such as cell shrinkage, membrane blebbing, nuclear condensation, and the formation of apoptotic bodies, and relies on the activation of caspases, a family of proteolytic enzymes that ensure that apoptosis proceeds in an orderly, often noninflammatory manner, facilitating tissue maintenance and immune tolerance [1,2].

In recent decades, several additional RCD pathways, including ferroptosis, pyroptosis, parthanatos, and autophagic cell death, have been identified. Each of these pathways involves unique molecular mechanisms and cellular outcomes and employs mechanisms distinct from those of apoptosis [1,3,4,5]. Among RCD mechanisms, ferroptosis, an iron-dependent process characterized by lethal lipid peroxidation that was first described by Stockwell and colleagues in 2012 [3], has emerged as particularly relevant to neurodegenerative disorders [6,7,8].

Ferroptosis differs fundamentally from other types of cell death in that it operates primarily through metabolic disruption rather than dedicated protein cascades. This process culminates in the oxidative destruction of cellular membranes through the convergence of three key mechanisms: disrupted antioxidant systems, dysregulated iron metabolism, and accelerated lipid peroxidation [9].

## 2. Significance and Scope

The brain presents a unique environment for iron homeostasis and ferroptosis regulation. Owing to its high oxygen consumption, abundant polyunsaturated fatty acids, and robust iron utilization for neurotransmitter synthesis and myelin formation, the central nervous system is especially vulnerable to ferroptotic damage. This vulnerability is further complicated by the blood-brain barrier (BBB), which tightly regulates iron influx and efflux, creating distinct compartmentalization of iron metabolism [10].

Neurodegenerative disorders represent a group of disorders characterized by the progressive loss of neuronal structure and function, manifesting as cognitive decline, motor dysfunction, and eventually neuronal death [11]. Major conditions include Alzheimer’s disease (AD), Parkinson’s disease (PD), Huntington’s disease (HD), multiple sclerosis (MS), and amyotrophic lateral sclerosis (ALS) [11]. These disorders share dysregulated iron homeostasis as a common feature.

Iron, which has long been recognized as a double-edged sword in biology, plays an indispensable role in central nervous system (CNS) functions and participates in multiple essential processes, such as oxygen transport, DNA synthesis, mitochondrial respiration, and neurotransmitter synthesis [12,13]. However, increased free iron can become neurotoxic, priming neurons for ferroptotic death [12,13].

Owing to their high metabolic demands, oxygen consumption, and lipid-rich membranes, neurons are especially vulnerable to oxidative damage. This vulnerability is exacerbated by the fine balance between the intracellular iron levels required for maintaining essential cellular functions and the conditions that promote iron overload [12,13]. When this balance is disrupted, the accumulation of free iron catalyzes harmful reactions that culminate in lipid peroxidation and ultimately results in ferroptotic cell death.

This vulnerability is further complicated by the blood-brain barrier (BBB), which tightly regulates iron influx and efflux, creating distinct compartmentalization of iron metabolism. Disruptions in BBB integrity, a common feature in many neurodegenerative conditions, can lead to aberrant iron accumulation within the brain parenchyma [10,14,15,16].

The importance of ferroptosis in neurodegenerative diseases extends beyond being merely a marker of pathology; it functions as an active mediator of disease progression. Under these conditions, characteristic patterns of iron accumulation precede or accompany neuronal degeneration, suggesting that iron-induced lipid peroxidation may be a critical upstream event driving pathological cascades.

Consequently, targeting ferroptosis regulatory pathways, whether by modulating iron homeostasis, enhancing antioxidant defenses, or inhibiting lipid peroxidation, represents a promising therapeutic strategy to mitigate the progressive neuronal loss characteristic of these diseases. However, despite considerable advances, significant knowledge gaps remain in our understanding of ferroptosis within the context of neurodegeneration. Our current understanding of this process is largely derived from studies in nonneuronal systems or simplified in vitro models, which fail to fully capture the complexity of neuronal iron metabolism or the unique microenvironment of the brain.

This review synthesizes current knowledge on the molecular mechanisms underlying ferroptosis and examines their roles in neurodegenerative disease pathology, with a particular focus on AD and PD, where ferroptosis has been best characterized. We further explored potential therapeutic strategies for modulating this cell death pathway. By addressing the molecular mechanisms of iron homeostasis, lipid peroxidation, and BBB function in the brain, we aim to provide insights that will guide future research and therapeutic development in the field of neurodegeneration.

## 3. Ferroptosis: Core Mechanisms and Iron Regulation

### 3.1. The System Xc^−^-GSH-GPX4 Regulatory Axis

**Figure 1:**
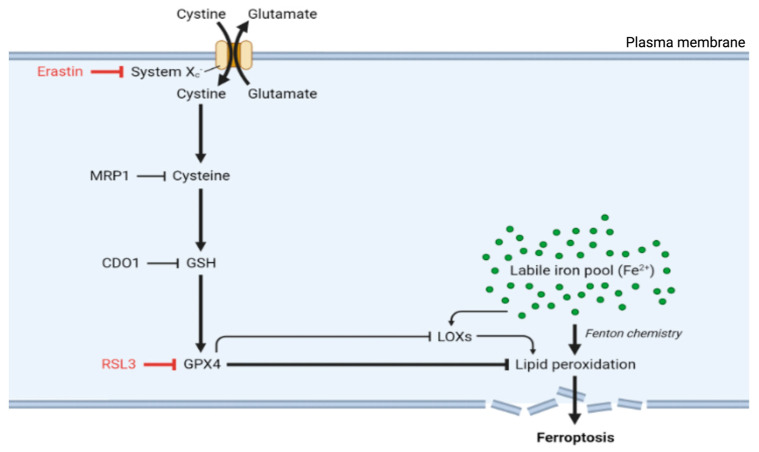
The system Xc^−^-GSH-GPX4 axis negatively regulates ferroptosis by suppressing excessive peroxidation of phospholipids. Lipid peroxidation is maintained by ferroptosis defense mechanisms mediated by GSH and GPX4, with GPX4 utilizing GSH to neutralize lipid peroxides, and cystine uptake mediated by system Xc^−^ provides the key precursor cysteine for GSH synthesis. The upregulation of either MRP1 or CDO1 counteracts this activity and decreases GSH synthesis. Suppression of system Xc^−^-GSH-GPX4 activity by the ferroptosis inducers erastin and RSL3 results in excessive lipid peroxidation, leading to membrane rupture and cell death. Abbreviations: CDO1, cysteine dioxygenase 1; GSH, reduced glutathione; GPX4, glutathione peroxidase 4; LOX, lipoxygenase; MRP1, multidrug-resistance-associated protein 1; RSL3, RAS-selective lethal. Created at https://BioRender.com (accessed on 10 March 2025).

Ferroptosis occurs through the convergence of three fundamental mechanisms: disrupted antioxidant systems, dysregulated iron metabolism, and accelerated lipid peroxidation [9]. The primary antioxidant defense against ferroptosis involves glutathione peroxidase 4 (GPX4). GPX4 acts as part of the system Xc^−^-GSH-GPX4 axis, which comprises the cystine/glutamate antiporter system Xc^−^, glutathione (GSH), and glutathione peroxidase 4 (GPX4) and is crucial for preventing ferroptosis. GPX4, a unique member of the oxidoreductase family, is a selenoenzyme that plays an essential role in protection against ferroptosis by converting toxic lipid hydroperoxides (L-OOH) into nontoxic lipid alcohols (L-OH) [9,17,18]. It utilizes glutathione (GSH) as a substrate for the reaction. GPX4 stands out among the glutathione peroxidase family as the sole enzyme capable of reducing complex lipid hydroperoxides within biological membranes. This unique capability makes GPX4 the primary defense against ferroptotic cell death, managing oxidative stress and maintaining membrane integrity by converting potentially lethal lipid hydroperoxides into nontoxic lipid alcohols (Figure 1) [19].

In addition to its direct antioxidant function, GPX4 also regulates the activity of lipoxygenases (LOXs), which are enzymes that promote the peroxidation of polyunsaturated fatty acids (PUFAs), underscoring the pivotal role of GPX4 as a key regulator of ferroptosis [20].

System Xc^−^, formed by the SLC7A11 and SLC3A2 subunits, functions as a cystine/glutamate antiporter that imports extracellular cystine in exchange for intracellular glutamate [21]. Once inside the cell, cystine is reduced to cysteine, an essential precursor for GSH synthesis. This GSH pool is the reducing substrate for GPX4, allowing it to detoxify lipid peroxides and inhibit ferroptosis. As a result, GSH levels, which are directly influenced by system Xc^−^ activity, are central to ferroptosis regulation [19].

When the protective action of the system Xc^−^-GSH-GPX4 protective axis fails, cells become vulnerable to ferroptotic death. For example, the inhibition of system Xc^−^ depletes cellular GSH reserves, whereas direct GPX4 inhibition permits the uncontrolled accumulation of lipid peroxides. Accordingly, treatment with erastin, a well-characterized ferroptosis inducer, blocks cystine uptake by inhibiting system Xc^−^, resulting in GSH depletion and ferroptosis (Figure 1) [3,22]. Furthermore, inducing GSH depletion through alternative routes can also be a catalyst for ferroptosis. Accordingly, the overexpression of multidrug-resistance-associated protein 1 (MRP1), which exports endogenous GSH, can sensitize cells to ferroptosis [23]. Similarly, the upregulation of cysteine dioxygenase 1 (CDO1) was shown to increase vulnerability to ferroptosis by inducing cysteine catabolism and, by extension, GSH depletion [24]. Moreover, direct inhibition of GPX4, whether via the use of the inhibitory molecule RAS-selective lethal (RSL3) or RNA interference (RNAi), also induces ferroptosis by allowing lipid peroxides to accumulate and damage cell membranes [25]. This GPX4 inhibition leads to an increase in reactive oxygen species (ROS) and promotes the conditions necessary for ferroptotic cell death [22].

The vulnerability of the system Xc^−^-GSH-GPX4 protective axis has significant implications for both disease mechanisms and therapeutic strategies, particularly in contexts where cells face high oxidative stress or increased metabolic demands [26,27,28,29]. On the one hand, therapeutic inhibition of the system Xc^−^-GSH-GPX4 axis is promising for inducing ferroptosis in cancer cells that have developed resistance to conventional apoptosis-inducing treatments. On the other hand, enhancing the system’s function could be beneficial in degenerative diseases where ferroptosis contributes to pathology, such as neurodegenerative diseases, which are mostly associated with oxidative stress and cell death.

While GPX4 was long thought to be the only enzyme that converts potentially lethal lipid hydroperoxides into nonlethal forms, genetic and chemical screens recently identified a complementary protective pathway. Ferroptosis suppressor protein 1 (FSP1, also known as AIFM2) defines a separate, parallel defense line that does not rely on GPX4 or glutathione [30,31,32]. Instead, FSP1 functions by reducing coenzyme Q10 (ubiquinone) to its antioxidant form, ubiquinol, which uses NADPH as an electron donor [30,31,32]. This regeneration of ubiquinol in the lipid bilayer prevents the propagation of lipid peroxidation independent of the GPX4/GSH axis. Thus, even when GPX4 is inhibited or GSH is depleted, FSP1 can maintain the redox integrity of membranes by limiting lipid peroxidation through the ubiquinone–ubiquinol cycle.

### 3.2. Lipid Peroxidation Drives Ferroptosis

Lipid peroxidation in ferroptosis follows a specific sequence of events beginning with the activation of polyunsaturated fatty acids (PUFAs). The enzyme ACSL4 first converts free PUFAs to acyl-CoA derivatives, which LPCAT3 then incorporates into membrane phospholipids. These membrane-integrated PUFAs become primary targets for iron-catalyzed peroxidation.

**Figure 2:**
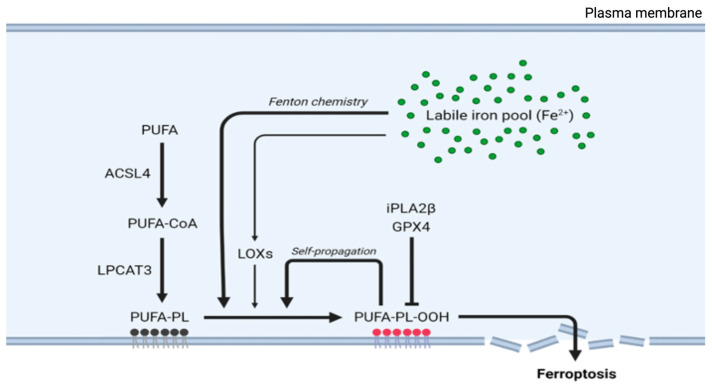
Lipid metabolism generates substrates for lipid peroxidation during ferroptosis. PUFA remodeling and incorporation into phospholipids through the actions of ACSL4 and LPCAT3 generate PUFA-PLs, the primary substrates of peroxidation in ferroptosis. The accumulation of lipid peroxides leads to profound cellular consequences, beginning with alterations in membrane properties. These changes include decreased membrane fluidity, disrupted membrane protein function, altered membrane organization, and, ultimately, membrane rupture and ferroptosis. Abbreviations: ACSL4, acyl-coenzyme A synthetase long-chain family member 4; GPX4, glutathione peroxidase 4; iPLA2β, calcium-independent phospholipase A2β; LPCAT3, lysophosphatidylcholine acyltransferase 3; LOX, lipoxygenase; PUFA, polyunsaturated fatty acid; PL, phospholipid. Created at https://BioRender.com (accessed on 10 March 2025).

The execution of ferroptosis requires the peroxidation of membrane-localized polyunsaturated fatty acids (FAs), a process that is enabled through the inhibition of GPX4 [6,33]. Nonetheless, compromised GPX4 function alone is insufficient for lipid peroxidation and subsequent ferroptosis; PUFAs must first be activated and incorporated into cellular membranes. In the initial phase, synthesized free PUFAs, including substances such as arachidonic acid, are transformed into acyl-coenzyme A thioesters (PUFA-CoAs) by the enzyme acyl-CoA synthetase long-chain family member 4 (ACSL4), a reaction necessary for PUFA utilization in membrane lipid synthesis (Figure 2) [34,35]. Subsequently, PUFA-CoAs undergo remodeling mediated by the enzyme lysophosphatidylcholine acyltransferase 3 (LPCAT3), thereby forming PUFA-containing phospholipids (PUFA-PLs), which are then incorporated into cell membranes [34,36]. Then, membrane-localized PUFA-PLs can be oxidized to generate toxic lipid peroxides (Figure 2).

Genetic screening of cells that are resistant to GPX4-inhibiting drugs suggested that ACSL4 and LPCAT3 are essential for ferroptosis triggered by the suppression of GPX4 activity [37]. In line with these findings, the inhibition of LPCAT3 by small-molecule inhibitors confers partial resistance to ferroptosis facilitated by GPX4 inhibition (Figure 2) [38]. Similarly, ACSL4-deficient cells exhibit resistance to ferroptosis upon knockout or inhibition of GPX4 [39]. On the other hand, the upregulation of ACSL4 has been linked to increased sensitivity to ferroptosis in different disease models [40,41,42,43,44,45].

The balance of lipid peroxidation is further modulated by calcium-independent phospholipase A2β (iPLA2β), which hydrolyzes peroxidized PUFA-PLs, thereby curtailing ferroptotic cell death [46]. This protective action highlights the importance of membrane-incorporated lipid peroxides as executors of ferroptosis; notably, supplementation with PUFA-PL peroxides, but not free PUFA peroxides, restores ferroptosis in ACSL4-deficient cells, demonstrating the critical role of membrane-localized lipid peroxides in ferroptosis [34,46,47].

In summary, the processes of PUFA remodeling and incorporation into phospholipids generate PUFA-PLs, the primary substrates of peroxidation in ferroptosis. The resulting membrane-localized lipid peroxides are the drivers of ferroptosis.

### 3.3. Reactive Oxygen Species (ROS)

The peroxidation process is accelerated through a self-propagating chain reaction. Iron, particularly in its ferrous (Fe^2+^) form, catalyzes the formation of ROS through the Fenton reaction [9], where ferrous iron (Fe^2+^) reacts with hydrogen peroxide to produce ferric iron (Fe^3+^) and hydroxyl radicals. These highly reactive hydroxyl radicals initiate the lipid peroxidation of polyunsaturated fatty acids (PUFAs) in cell membranes [48,49]. Lipid peroxides are unstable molecules that can decompose into various reactive aldehydes, further amplifying damage to cellular components and enhancing ROS production. The main sources of ROS are enzymes such as lipoxygenases (LOXs) and cytochrome P450 enzymes, which are key in the enzymatic production of ROS during ferroptosis by contributing to the formation of lipid hydroperoxides from PUFAs [50].

The ROS generated in ferroptosis propagate a chain reaction of lipid peroxidation, which leads to further ROS production and cellular damage. This propagation can be self-amplifying unless interrupted by lipophilic ROS-trapping antioxidants such as vitamin E or inhibitors of lipid peroxidation, such as ferrostatins or liproxstatins [51,52,53]. Furthermore, the inactivation of GPX, which catalyzes the reduction of lipid hydroperoxides to nontoxic alcohols, enhances susceptibility to ferroptosis [54].

Ultimately, the reactive species attack membrane-bound PUFAs, generating lipid radicals that perpetuate the peroxidation cascade [7,8,55]. This process specifically targets membrane phospholipids containing arachidonic and adrenic acids, explaining why cells enriched with these fatty acids show increased susceptibility to ferroptosis.

The final stages of ferroptosis involve catastrophic membrane failure leading to cell death. Unlike the controlled membrane blebbing observed in apoptosis, ferroptotic cell death results from widespread membrane peroxidation and subsequent rupture. This process can trigger inflammatory responses in surrounding tissue, as cellular contents leak into the extracellular space.

### 3.4. Mitochondrial Contribution to Ferroptosis

In addition to their function in energy production, mitochondria play crucial roles in ferroptosis [56,57]. During normal respiratory function, electrons occasionally leak from complexes I and III, leading to the partial reduction of oxygen and the formation of superoxide radicals. NADPH oxidases (NOX), membrane-bound enzymes that are capable of transferring electrons from NADPH to oxygen, can provide an additional source of ROS, particularly under stress conditions [57]. Furthermore, ferroptotic stress induces a significant shift in energy production with increased oxidative phosphorylation and ATP generation, whereas glucose metabolism through glycolysis decreases. This causes a surge in ROS formation and oxidative stress, leading to permanent mitochondrial damage [57].

As mitochondrial membranes suffer oxidative damage, the ETC becomes increasingly dysfunctional, leading to further electron leakage and ROS production [56,57]. This vicious cycle is exacerbated by the presence of free iron, which catalyzes the Fenton reaction, generating highly reactive hydroxyl radicals. The mitochondrial iron pool, which is normally tightly regulated for heme synthesis and iron-sulfur cluster assembly, becomes labile during ferroptosis, driving ROS production and contributing to energy failure [57].

Mounting evidence suggests that the mitochondrial control of ferroptosis through iron homeostasis, ROS production, and metabolic regulation is particularly important for neurodegenerative processes.

The impact of ferroptosis on mitochondria extends beyond ROS generation to the balance of mitochondrial fusion and division and the removal of damaged mitochondria through mitophagy, which makes neurons particularly vulnerable to ferroptosis.

## 4. Iron Homeostasis and Ferroptosis Regulation

Iron homeostasis represents a critical control point in ferroptotic cell death and involves tightly coordinated regulation of iron import, storage, utilization, and export pathways. Each of these processes is controlled by specific proteins and regulatory factors that collectively maintain appropriate iron levels while preventing excessive accumulation.

The intracellular metabolism of iron can be roughly categorized into the uptake, storage, utilization, and export of iron—each orchestrated by regulatory proteins to maintain the cellular redox balance and avoid oxidative damage.

### 4.1. Regulation of Iron Uptake

**Figure 3:**
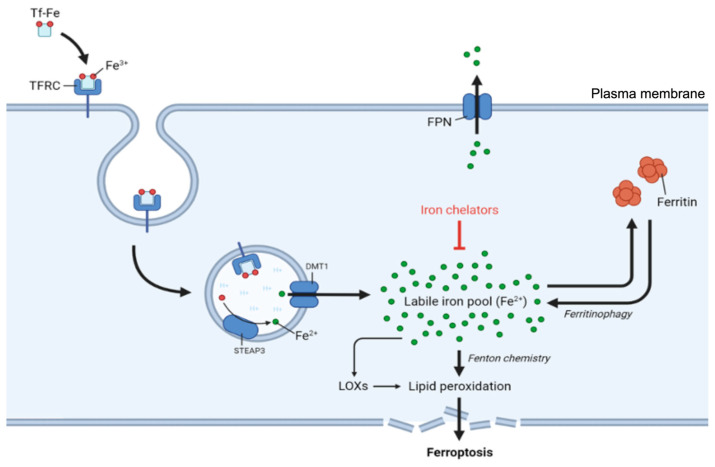
Iron metabolism facilitates lipid peroxidation in ferroptosis. Extracellular Fe^3+^ attached to transferrin (Tf) binds to the transferrin receptor, leading to the internalization of the transferrin–receptor complex (TFRC). Once inside the endosome, STEAP3, a metalloreductase, converts Fe^3+^ into Fe^2+^. DMT1 then shuttles Fe^2+^ from the endosome to a cytoplasmic labile iron pool, where Fe^2+^ generates ROS through the Fenton reaction. Fe^2+^ can also be exported via the FPN. Abbreviations: DMT1, divalent metal transporter 1; FPN, ferroportin; LOX, lipoxygenase; ROS, reactive oxygen species; STEAP3, six-transmembrane epithelial antigen of the prostate 3; Tf, transferrin; TFRC, transferrin–receptor complex. Created at https://BioRender.com (accessed on 10 March 2025).

Iron import primarily occurs via transferrin receptor 1 (TfR), which binds to transferrin-bound iron on the cell surface [58]. Iron in the blood is bound by transferrin (Tf), a natural chelating plasma protein. When iron-laden transferrin binds to TfR on the cell surface, the resulting complex (TFRC) undergoes clathrin-mediated internalization into endosomes (Figure 3). Within the endosome, the lower pH of the environment prompts iron (Fe^3+^) to dissociate from transferrin and undergo reduction from Fe^3+^ to Fe^2+^, which is catalyzed by the ferrireductase STEAP3. The reduced iron is then transported across the endosomal membrane into the cytosol by divalent metal transporter 1 (DMT1) [58,59], while the transferrin–TfR complex is recycled back to the cell surface for additional rounds of iron uptake.

TfR1 recycling to the cell surface is mediated by the endosomal sorting machinery, particularly the retromer complex, in association with the adaptor sorting nexin 3 (SNX3) [60,61]. This complex recognizes specific motifs on TfR1 and facilitates its return to the plasma membrane. Disruption of this recycling pathway can alter cellular iron uptake and has been implicated in altered ferroptosis sensitivity. Accordingly, SNX3 overexpression confers increased ferroptosis sensitivity, whereas SNX3 loss confers ferroptosis resistance [62]. Consequently, the accumulation of TfRs on the plasma membrane has been established as an accurate marker of ferroptosis [63].

Another way in which cells regulate the intracellular concentration of iron is by modulating the abundance of TfRs by altering the expression of TfRs. For example, during iron deficiency, iron regulatory proteins (IRPs, see below) can promote increased TfR expression by stabilizing TfR mRNA [64].

### 4.2. Regulation of Iron Storage and Mobilization

**Figure 4:**
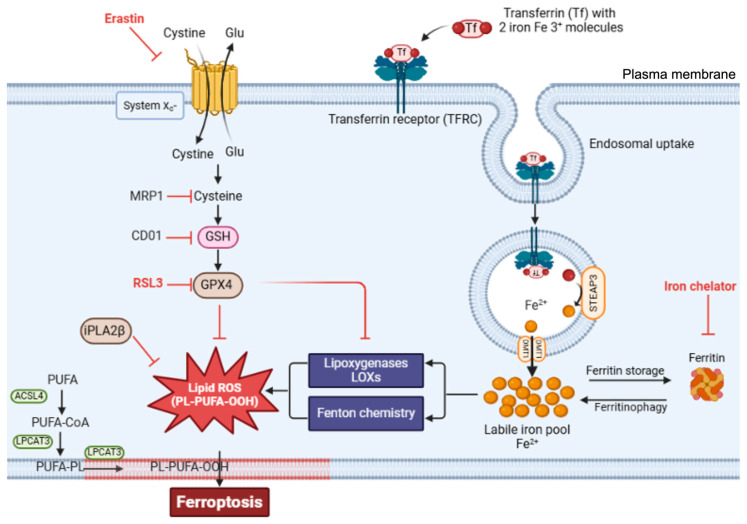
Summary of the key mechanism underlying ferroptotic cell death. Ferroptosis is primarily governed by three interrelated pathways: the GSH/GPX4 antioxidant system, iron metabolism, and lipid peroxidation. The initiation of ferroptosis is driven by two critical events: suppression of the SLC7A11/GSH/GPX4 axis and the accumulation of intracellular free iron. Iron is imported into cells via transferrin-mediated endocytosis after binding to the transferrin receptor (TFR), forming the TFR complex (TFRC). Once inside the cell, ferric iron (Fe^3+^) is converted to ferrous iron (Fe^2+^) and transported into the cytoplasm via the action of STEAP3 and DMT1, respectively. Fe^2+^, once imported into the cytoplasm, either enters the labile iron pool, where it participates in redox reactions such as the Fenton reaction, or is sequestered and stored in a redox-inactive form by ferritin to prevent oxidative damage. The accumulation of redox-active iron promotes the formation of lipid peroxides and thus contributes to ferroptotic cell death. The system Xc^−^ antiporter imports extracellular cystine in exchange for intracellular glutamate at a 1:1 ratio. Intracellularly, cystine is reduced to cysteine, which serves as a precursor for glutathione (GSH) synthesis. This process is catalyzed by glutathione synthase (GSS). Glutathione peroxidase 4 (GPX4) plays a central role by reducing lipid hydroperoxides (PL-OOH) to nontoxic lipid alcohols (PL-OH) using GSH as a reducing agent. The lipid composition of cellular membranes also determines susceptibility to ferroptosis. Long-chain fatty acyl-CoA synthetase 4 (ACSL4) and lysophosphatidylcholine acyltransferase 3 (LPCAT3) promote the incorporation of polyunsaturated fatty acids (PUFAs) into membrane phospholipids, generating PUFA-containing phospholipids (PUFA-PLs), which are highly susceptible to oxidation by reactive oxygen species (ROS), thereby promoting lipid peroxidation and ferroptosis. Several chemical and genetic modulators influence ferroptotic sensitivity. Iron chelators such as deferoxamine inhibit ferroptosis by reducing the labile iron pool and preventing iron-catalyzed oxidative damage. Erastin promotes ferroptosis by inhibiting system Xc^−^, leading to cystine and glutathione depletion and impaired GPX4 function. Similarly, RSL3 directly inhibits GPX4, resulting in lipid peroxide accumulation. Multidrug-resistance-associated protein 1 (MRP1) exacerbates ferroptosis by exporting intracellular glutathione, diminishing antioxidant capacity. Cysteine dioxygenase 1 (CDO1) shifts cysteine metabolism away from glutathione synthesis toward taurine production, thereby reducing cellular defense against oxidative stress. In contrast, calcium-independent phospholipase A2 beta (iPLA2β) acts as a suppressor of ferroptosis by hydrolyzing oxidized phospholipids and attenuating lipid peroxidation. Created via BioRender.com (accessed 1 December 2024).

Once in the cytosol, iron exists either as part of the labile iron pool or in storage complexes. The labile iron pool, which consists of redox-active Fe^2+^, requires strict regulation because of its potential to generate harmful ROS through Fenton chemistry (Figure 4). To prevent excessive accumulation of free iron, cells utilize the iron storage protein ferritin [65,66], which can sequester thousands of iron atoms in a relatively inert form.

The ferritin complex, which is composed of 24 subunits of heavy (FTH) and light (FTL) chains, plays a central role in iron storage [66]. FTH possesses ferroxidase activity that converts Fe^2+^ to Fe^3+^, whereas FTL provides nucleation sites for mineralized iron core formation [66]. The ratio of FTH to FTL varies among tissues and can be modulated in response to oxidative stress and inflammation, allowing cells to fine-tune their iron storage capacity and antioxidant defenses.

The regulation of iron storage critically impacts ferroptosis: ferroptosis sensitivity can be significantly influenced by agents that reduce iron availability, such as the iron chelator deferoxamine, as well as by changes in iron-regulating proteins that modulate the cellular labile iron pool [3]. For example, ferroptosis sensitivity can be increased by overexpression of TfR, which enhances iron uptake, and by decreased expression of FTH1 and FTL, which lowers ferritin levels and thereby reduces iron storage [25].

Ferritinophagy, the selective autophagic degradation of ferritin, represents an additional regulatory mechanism in cellular iron homeostasis and ferroptosis sensitivity [67]. This process involves nuclear receptor coactivator 4 (NCOA4), which functions as a selective cargo receptor that recognizes and delivers ferritin to form autophagosomes [67].

The interaction between NCOA4 and ferritin is regulated by cellular iron levels, with high iron concentrations promoting NCOA4 degradation through an iron-dependent ubiquitination mechanism mediated by the E3 ubiquitin ligase HERC2 [67,68,69]. Under conditions of iron deficiency, increased NCOA4-mediated ferritinophagy helps maintain adequate levels of bioavailable iron by releasing it from ferritin stores [69,70]. Conversely, oxidative stress can modulate ferritinophagy through effects on the autophagy machinery and NCOA4 stability [70].

The role of ferritinophagy in ferroptosis is significant, as increased ferritinophagy can promote ferroptotic cell death by expanding the labile iron pool. Inhibition of NCOA4 can protect cells from ferroptosis by preventing the release of iron from ferritin stores, whereas conditions that increase ferritinophagy can sensitize cells to ferroptotic death [71,72].

### 4.3. Iron Regulatory Network

Iron homeostasis is further regulated through an iron regulatory network that adjusts iron-dependent gene expression through iron regulatory proteins (IRPs) [64]. IRPs respond to the cellular iron status by controlling the expression of iron transport, storage, and export proteins through interactions with iron-responsive elements (IREs) in their mRNAs [64]. IRP1 contains an iron-sulfur cluster that, when assembled under iron-replete conditions, converts the protein into cytosolic aconitate hydratase, preventing IRE binding [64]. In contrast, IRP2 is regulated through iron-dependent proteasomal degradation mediated by the E3 ubiquitin ligase FBXL5, which acts as a direct iron sensor through its hemerythrin domain [64].

When cellular iron levels are low, IRPs bind to IREs in the untranslated regions (UTRs) of target mRNAs. The position of the IRE determines the regulatory outcome: IRP binding to 5′ UTR IREs blocks translation initiation, whereas binding to 3′ UTR IREs stabilizes the mRNA [64]. This dual mechanism allows the coordinated regulation of iron metabolism proteins. For example, under low-iron conditions, IRPs simultaneously repress the translation of ferritin and ferroportin (5′ IRE) while stabilizing transferrin receptor (3′ IRE) mRNA, thereby promoting iron acquisition while preventing iron storage and export.

### 4.4. Regulation of Iron Export

Iron export represents another important regulatory point and is mediated by ferroportin (SLC40A1), the sole known cellular iron exporter in vertebrates. This transmembrane protein, encoded by *FPN1*, facilitates the translocation of ferrous iron (Fe^2+^) across the plasma membrane, working in concert with ferroxidases that convert the exported Fe^2+^ to Fe^3+^ for safe transport in the circulation [73].

Ferroportin is regulated at the systemic level by the peptide hormone hepcidin, which binds directly to ferroportin and its ubiquitination, internalization, and subsequent lysosomal degradation effectively block cellular iron export [74]. At the cellular level, ferroportin expression is controlled by the IRP/IRE system, with IRPs repressing ferroportin translation under low-iron conditions [65,75].

Hepcidin is produced primarily by the liver in response to high iron levels, inflammation, or other physiological signals and provides rapid control over cellular iron export, allowing a quick response to changing physiological conditions or environmental challenges [76,77]. Blockage of hepcidin-mediated regulation of ferroportin, therefore, can lead to disorders caused by iron overload [78].

Alterations in ferroportin expression or function can significantly impact cellular iron levels and subsequent susceptibility to ferroptotic cell death. For example, inflammatory signals can suppress ferroportin transcription during infection or inflammation, sensitizing cells to ferroptosis through intracellular iron accumulation [65]. Conversely, increased ferroportin expression or activity may protect against ferroptosis by reducing cellular iron levels, although this protection must be balanced against the requirements for essential iron-dependent cellular processes [65].

## 5. Brain-Specific Iron Homeostasis: The Critical Role of the Blood-Brain Barrier

### 5.1. Iron Functions in the Central Nervous System

Iron plays crucial roles in the CNS by sustaining neuronal energy metabolism and facilitating neural communication and plasticity [12]. It serves as an essential cofactor for enzymes involved in neurotransmitter synthesis, including tyrosine hydroxylase for dopamine production and tryptophan hydroxylase for serotonin synthesis [12]. Iron is also vital for myelination, as oligodendrocytes require iron for their development and for the production of myelin components [12].

Despite these essential functions, the high oxygen consumption and lipid-rich composition of the CNS render it particularly vulnerable to iron-induced oxidative damage [12]. Consequently, the brain has evolved specialized mechanisms to regulate iron uptake, distribution, and storage, with the blood-brain barrier (BBB) serving as the primary interface for controlled iron transport between systemic circulation and brain tissue.

### 5.2. Structure and Function of the Blood-Brain Barrier in Iron Transport

**Figure 5:**
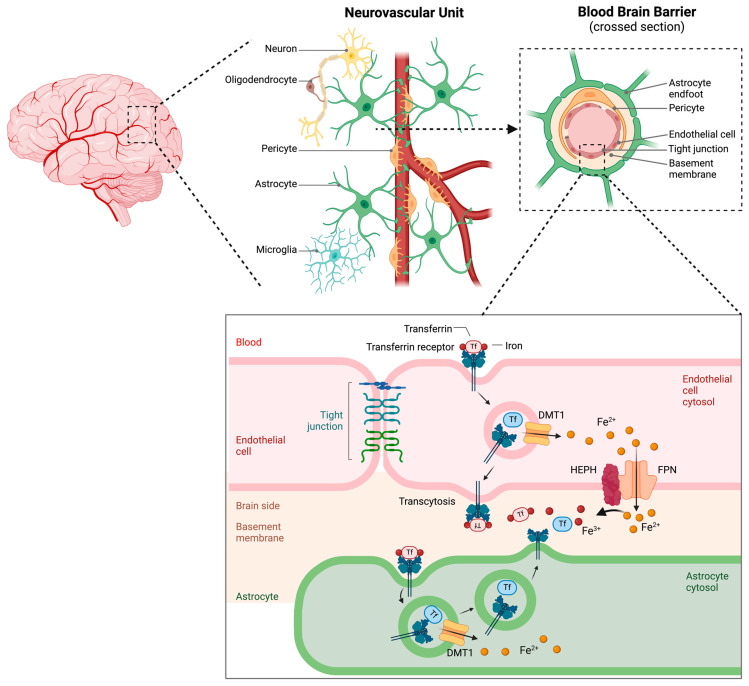
Model depicting the neovascular unit of the brain with a cross-section delineating the arrangement of cell types forming the blood-brain barrier (BBB). A magnified image of a section of the BBB is shown, depicting two capillary endothelial cells connected by tight junctions along with a section of an astrocyte end-foot. The main routes of iron uptake by capillary endothelial cells involve three main processes: uptake by endocytosis, transcytosis, and FPN-mediated export. Initially, transferrin (Tf, red), which is iron-bound, binds to the transferrin receptor (TfR) on the luminal membrane of endothelial cells, and the resulting transferrin–receptor complex (TFRC) is subsequently endocytosed. Following internalization, the complex can proceed via two distinct routes. In the first route, termed transcytosis (1), the TFRC is directly shuttled to the abluminal membrane, where Tf is then released into the extracellular fluid of the brain. Alternatively, the TFRC is dissociated within the acidic environment of the endosome. In this case, ferric (Fe^3+^) iron is released from Tf and reduced to ferrous (Fe^2+^) iron via the action of STEAP proteins, yielding apo-transferrin (apo-Tf, blue). The apo-Tf is then recycled back to the luminal membrane along with the TfR. Ferrous (Fe^2+^) iron is subsequently released into the cytoplasm via DMT1, where it can be exported via the ferroportin (FPN) export pathway (2). Hephaestin (HEPH), a membrane-bound homologue of ceruloplasmin, acts as a ferroxidase, converting Fe^2+^ to ferric (Fe^3+^) iron to facilitate iron export via ferroportin. The action of HEPH enables apo-Tf to bind iron for utilization by other cells. Created at https://BioRender.com (accessed on 12 March 2025).

The BBB is a highly specialized, selectively permeable barrier composed primarily of brain microvascular endothelial cells joined by tight junctions alongside pericytes, astrocytic end-feet, and a supporting basement membrane (Figure 5) [10]. The BBB plays a central role in cerebral iron homeostasis by tightly regulating iron trafficking between the circulation and brain parenchyma [10].

Systemic iron originates primarily from dietary sources and is absorbed in the duodenum [79]. Once in circulation, it binds to transferrin, which prevents excessive redox activity in the bloodstream. Iron uptake across the BBB primarily occurs through TfR-mediated endocytosis. Brain microvascular endothelial cells import transferrin-bound iron from the blood and export iron into the brain interstitium through the iron exporter ferroportin. These cells express high levels of TfR1 on their luminal side, which mediates the uptake of transferrin-bound iron from the circulation through endocytosis [80,81,82]. Within acidic endosomes, iron is released from transferrin and reduced from Fe^3+^ to Fe^2+^, which is often facilitated by STEAP proteins [80,81,82]. The reduced iron is then exported into the cytosol via DMT1 and subsequently transported into the brain parenchyma through ferroportin (Figure 5) [10,14,80,83]. Ferrous iron (Fe^2+^) is then converted to ferric iron (Fe^3+^) through the action of ferroxidases such as hephaestin (HEPH) or ceruloplasmin to facilitate iron export via ferroportin (Figure 5) [14,84,85,86]. By oxidizing ferrous iron to the ferric state, HEPH enables exported iron to bind transferrin for transport. This function is analogous to that of ceruloplasmin in the circulation and brain: both HEPH and ceruloplasmin ensure that cellular iron release does not lead to toxic ferrous accumulation. Consistent with this role, when ceruloplasmin is lacking in astrocytes, neurons increase HEPH expression to maintain iron efflux, preventing intracellular iron build-up [87]. In Figure 5, HEPH is thus depicted as partnering with ferroportin at the cell membrane to promote safe iron export, essentially substituting for ceruloplasmin’s ferroxidase activity at the interface of cells and extracellular fluids.

Ion uptake into the brain parenchyma is controlled by the peptide hormone hepcidin, analogous to its role in systemic iron regulation. Hepcidin binding causes internalization and degradation of ferroportin, thereby limiting iron efflux into the brain. Indeed, astrocyte-derived hepcidin has been shown to act on BBB endothelial ferroportin to restrict iron entry; experimentally, ventricular administration of hepcidin reduces iron influx into brain tissue and ameliorates iron overload [88]. Conversely, hepcidin deficiency in the brain leads to increased endothelial ferroportin and excessive iron accumulation in neural tissues, with accompanying cognitive impairment [88]. Thus, the BBB–hepcidin axis serves as a critical checkpoint in preventing iron dysregulation within the central nervous system.

To meet increased iron demands or respond to fluctuations, particularly under conditions of stress, the CNS can also engage nontransferrin-bound iron uptake pathways [14]. These alternative mechanisms include the involvement of lactoferrin receptors and other transporters capable of binding nontransferrin-bound iron.

### 5.3. Iron Distribution and Regulation in the Brain Parenchyma

Once in the brain parenchyma, iron is distributed among neurons, astrocytes, microglia, and oligodendrocytes, each with distinct iron requirements and regulatory mechanisms [12,14]. Neurons express TfR, STEAP, and DMT1 for iron uptake, and some neuronal populations also express ferroportin for iron export [89].

Astrocytes play dual roles in brain iron homeostasis, both by storing iron in ferritin and supplying iron to neurons as needed [90]. They express both iron importers and exporters, allowing them to respond dynamically to changes in local iron demand [12,14,90]. Oligodendrocytes, which are responsible for myelin production, contain high levels of iron and express ferritin for iron storage [12,14,90]. The high iron requirement of these cells makes them particularly vulnerable to iron-mediated oxidative stress and ferroptosis.

Microglia, as resident immune cells of the CNS, can phagocytose damaged cells and degrade iron-containing proteins, thereby contributing to iron recycling within the brain [12,14]. During neuroinflammation, activated microglia can release stored iron, potentially contributing to oxidative stress in the surrounding tissue [12,14].

The regulation of iron within the brain parenchyma involves cell-specific expression patterns of iron transporters, storage proteins, and regulatory factors, allowing fine-tuned control of local iron concentrations. Iron efflux from the brain may occur via ferroportin-mediated export back across the BBB [91,92] or through the glymphatic system [93], which contributes to the clearance of metabolic waste from the brain.

Taken together, key mediators determining iron levels in the brain include TfR, which can increase neuronal iron uptake if overexpressed; ferroportin, whose reduced expression can impede iron export; and ferritin, which serves as the major iron storage protein but may be excessively degraded through ferritinophagy. Dysregulation of any of these control points can promote iron overload in the brain.

## 6. Disruption of Iron Homeostasis in Neurodegenerative Diseases

### 6.1. Common Pathways of Iron Dysregulation in Neurodegeneration

Neurodegenerative diseases share common pathways of iron dysregulation despite their distinct clinical and pathological features [12,94]. These include BBB dysfunction, altered expression of iron transporters and storage proteins, impaired iron export mechanisms, and disrupted cellular iron handling.

BBB dysfunction, a feature of many neurodegenerative conditions, or altered expression of iron transporters (e.g., increased TfR1 or DMT1 levels) can lead to unregulated iron influx into the brain parenchyma [10].

Within the brain parenchyma, dysregulated expression of iron regulatory proteins is commonly observed [12,94]. Increased expression of iron import proteins, coupled with decreased expression of storage proteins or export mechanisms, can lead to iron accumulation in vulnerable regions and, potentially, promote ferroptosis.

Additionally, neuroinflammation, a hallmark of neurodegenerative diseases, and age-related changes in iron metabolism can exacerbate iron overload and trigger ferroptosis [95,96].

The role of iron overload and ferroptosis in disease pathogenesis has been most extensively studied in AD and PD, revealing both shared and unique characteristics that suggest that ferroptosis is a potential driver of cell death. Below, we examine the evidence linking iron dyshomeostasis (through BBB disruption and altered iron handling in the brain) and iron-mediated neuronal cell death (influenced by mitochondrial ROS, neuroinflammation, and aging) to the disease pathology of AD and PD.

### 6.2. Blood-Brain Barrier Dysfunction in Alzheimer’s and Parkinson’s Disease

Dysfunction of the BBB is an important contributing factor in neurodegenerative diseases. BBB breakdown through the disruption of tight junctions between endothelial cells potentially allows unregulated paracellular iron influx, bypassing normal regulatory mechanisms [97].

In AD, the breakdown of tight junction complexes has been identified as a key feature of BBB dysfunction [98,99,100]. Studies using human postmortem tissues confirmed selective reductions in tight junction protein levels in cortical regions of AD brains, which correlated with synaptic degeneration and amyloid-beta (Aβ) accumulation [99,101]. Importantly, BBB disruption was shown to be an early marker of cognitive impairment in AD, regardless of Aβ or tau pathology or other signs of vascular disease [102].

Changes in BBB permeability in AD are also linked to dysregulated transport mechanisms, which facilitate Aβ influx into the brain and impair Aβ clearance [103,104]. Indeed, Aβ may further modulate iron transport across the BBB. Accordingly, Aβ-exposed astrocytes stimulate an increase in endothelial TfR1 and DMT1 expression in patients’ brain regions vulnerable to AD pathology, such as the hippocampus and cortex, thereby potentially increasing transferrin-mediated iron uptake by endothelial cells [105].

Additionally, genetic factors, particularly the apolipoprotein E (APOE) ε4 allele, have been strongly implicated in BBB breakdown in AD [100,106]. APOEε4 carriers exhibit increased BBB permeability in vitro and in vivo, as demonstrated through diverse approaches, including MRI-based imaging, biomarker analyses, and an induced pluripotent stem cell (iPSC) three-dimensional model, whereas studies on postmortem brain tissue have revealed pericyte loss or vascular degeneration linked to APOEε4-driven neuroinflammation [106,107].

BBB dysfunction in PD is less characterized. While tight junction disruption and transporter remodeling are well defined in AD, parallel investigations into PD remain underdeveloped, with fewer human studies examining tight junction protein loss or transporter dysfunction in relation to α-synuclein pathology [97,108]. iPSC-based models suggest that familial PD mutations may compromise BBB integrity [109]; however, direct human data remain sparse. Furthermore, inflammation-driven BBB disruption appears to be a shared feature between AD and PD, with elevated cytokine and oxidative stress responses contributing to vascular damage in both diseases, although the specific molecular pathways involved remain better characterized in AD [98,100,110].

In summary, aberrantly regulated iron uptake mechanisms at the BBB play important roles in the pathogenesis of AD and, potentially, PD. The dysregulation of TfR1, DMT1, and other iron transporters, in conjunction with BBB compromise and neuroinflammatory signals, may potentiate oxidative stress and thereby render neurons particularly susceptible to ferroptotic cell death.

### 6.3. Iron Homeostasis and Ferroptosis Susceptibility in Alzheimer’s and Parkinson’s Disease

Iron dyshomeostasis is increasingly recognized as a significant contributor to AD pathogenesis, with multiple lines of evidence showing disrupted iron regulation in affected brain regions, particularly those crucial for memory and cognition [111].

Amyloid precursor protein (APP) has been implicated in the modulation of iron export through interactions with ferroportin [112,113]. Studies have shown that APP facilitates iron export by interacting with ferroportin, particularly in cells lacking ceruloplasmin, such as cortical neurons [113]. The absence of APP leads to iron retention and oxidative stress, highlighting its role in iron metabolism. Notably, APP possesses ferroxidase activity, similar to ceruloplasmin, which is essential for oxidizing Fe^2+^ to Fe^3+^ before it can be exported via ferroportin [113]. This activity is important for loading iron onto transferrin and preventing oxidative stress. The alteration in APP processing observed in AD may disrupt this function, contributing to iron accumulation. Furthermore, as described in more detail below, Aβ plaques and tau pathology intersect with iron homeostasis [114,115,116], as these protein aggregates can bind iron, creating local concentrations of redox-active iron that promote oxidative stress.

Genetic factors associated with AD risk are also mechanistically connected to iron homeostasis. The APOE ε4 allele, the strongest genetic risk factor for late-onset AD, has been associated with increased brain iron accumulation in human patients and significantly elevated ferritin levels in their cerebrospinal fluid, which is associated with accelerated AD pathology [117,118,119]. Variants in other genes involved in iron transport and storage have been identified as potential modifiers of AD risk and progression [120]. Furthermore, in mice, APOE is required for iron homeostasis in the brain because it modulates TfR1, IRPs, Fpn1, aconitase, and hepcidin in the hippocampus and basal ganglia [121].

Like AD, iron overload is a well-established feature of PD. However, iron overload in PD is predominantly associated with α-synuclein aggregation, neuroinflammation, and mitochondrial dysfunction [122,123]. The SNpc, the brain area most vulnerable to neuron loss in PD, is naturally rich in iron [124,125]. This selective vulnerability reflects the unique characteristics of dopaminergic neurons, including their high iron requirements for dopamine synthesis, the presence of neuromelanin that can bind iron, their high polyunsaturated fatty acid content, and their elevated metabolic rate [126,127,128,129,130]. These characteristics create an environment where even small disruptions in iron homeostasis can have significant consequences [131,132,133].

Furthermore, dopamine metabolism itself contributes to the vulnerability of SNpc neurons to iron-mediated damage. Dopamine autooxidation generates ROS and quinones, which can interact with iron to increase oxidative stress [123]. Additionally, dopamine metabolism through monoamine oxidase produces hydrogen peroxide as a byproduct [123], which can participate in mitochondrial iron-catalyzed Fenton reactions [134].

PD brains, like AD brains, also show altered expression of iron regulatory proteins. Increased expression of TfR1 and DMT1 has been observed in the SNpc of PD animal models and PD patients [135], potentially enhancing iron uptake. Studies also report reduced expression of ferroportin, which impairs iron export from neurons [136]. Additionally, changes in ferritin expression and increases in iron-responsive-element-binding proteins have been documented [137], reflecting disrupted iron storage and regulation.

Furthermore, genetic factors associated with PD are significantly associated with iron homeostasis and increased susceptibility to ferroptosis. Mutations in several PD-associated genes, including LRRK2, PINK1, Parkin, and DJ-1, affect mitochondrial function and iron metabolism [138,139,140]. For example, PINK1 and Parkin mutations can disrupt mitochondrial iron handling and increase oxidative stress, potentially increasing susceptibility to ferroptotic cell death [141,142,143,144,145,146,147,148,149,150]. DJ-1 mutations may compromise cellular antioxidant defenses, increasing the vulnerability of neurons to iron-dependent oxidative damage [138,151,152,153].

Taken together, the combination of elevated baseline iron levels and disease-related dysregulation of iron metabolism may create a toxic environment that underlies the selective vulnerability of dopaminergic neurons in PD. Indeed, increased mitochondrial oxidant stress in human SNc neurons has been shown to initiate a dopamine- and iron-dependent toxic cascade, leading to lysosomal dysfunction and α-synuclein accumulation in the presence of a PD-associated DJ-1 mutation—thus establishing a causal link among key pathological features of PD [154,155].

In summary, both AD and PD patients exhibit altered expression of iron transport and storage proteins in the brain. Specifically, increased DMT1 and TfR1 expression in vulnerable regions may enhance iron uptake, while decreased ferroportin expression can impair iron export, further promoting iron accumulation. Additionally, changes in ferritin levels and composition may reflect disrupted iron storage capacity. Finally, disease-specific factors and genetic mutations may contribute to multiple pathological mechanisms across these disorders.

## 7. Linking Iron Dysregulation, Ferroptosis, and Neurodegeneration

### 7.1. Regional Selectivity of Iron Accumulation and Neuronal Vulnerability

A striking feature of neurodegenerative diseases is their regional selectivity, with specific brain areas showing greater vulnerability to pathology. This selectivity coincides with patterns of iron accumulation, suggesting a relationship between regional iron handling and disease progression. 

Neuroimaging studies using techniques such as quantitative susceptibility mapping and magnetic resonance imaging have consistently revealed elevated iron levels in critical regions [156,157,158]. In AD, iron accumulation is particularly pronounced in the hippocampus, associated cortices, and certain subcortical nuclei [158], regions critical for memory and cognition. This pattern aligns with the distribution of tau pathology and neurodegeneration. Importantly, these changes can be detected even in the prodromal or mild cognitive impairment (MCI) stages, supporting the notion that iron dysregulation is likely an early event in AD pathogenesis [159,160].

PD shows a distinct pattern of iron accumulation primarily centered on the SNpc [128,161], which coincides with the primary site of dopaminergic neuron loss [131,132,133]. Similar to AD, even in the early stages of PD, iron metabolism is disturbed, and iron begins to redistribute in the brain, particularly in regions such as the SNpc [162,163,164].

Taken together, although ferroptosis is implicated in the neurodegeneration of both AD and PD, the underlying mechanistic details differ between these diseases. A key difference lies in the regional distribution of iron and affected cells. In PD, excess iron accumulates predominantly in the substantia nigra, and ferroptotic injury is largely confined to the vulnerable dopaminergic neurons in this region. These neurons, enriched in neuromelanin-bound iron, are uniquely predisposed to iron-catalyzed lipid peroxidation and undergo ferroptosis when antioxidant defenses fail. In contrast, in AD, iron dysregulation is more widespread across the cortex and hippocampus, where elevated iron levels are associated with faster cognitive decline. Accordingly, ferroptosis in AD may affect a broader array of cells.

Not only are neurons in high-iron areas susceptible to ferroptotic death, glial cells also contribute. Activated microglia in AD often sequester iron (for example, around amyloid plaques) and can themselves undergo ferroptosis [165]. Notably, iron-overloaded microglia can amplify neurodegeneration by releasing oxidative metabolites and inflammatory mediators upon ferroptotic stress (see also Section 7.5), thereby harming nearby neurons. Thus, PD manifests a more neuron-selective ferroptosis, primarily targeting nigral dopaminergic neurons, whereas AD is more complex with both neurons and iron-loaded glia (microglia, and possibly astrocytes) participating in ferroptotic pathways.

The distinct regional accumulation of iron in AD and PD patients may reflect differences in baseline iron content, variations in the expression of iron regulatory proteins, or region-specific responses to aging and inflammation. Localized iron leakage across a compromised BBB may generate ROS in affected regions, which further damages BBB integrity, creating a detrimental feedback loop. Notably, BBB dysfunction does not occur uniformly across the brain: the hippocampus and certain cortical regions exhibit an early and more severe BBB breakdown in patients with AD [102]. As described earlier, this breakdown is associated with cognitive impairment and can occur independently of Aβ pathology [102]. Similarly, the midbrain, including the substantia nigra, also exhibits a localized increase in BBB permeability in patients with PD [166].

Overall, the distinctions in iron overload and cell death susceptibility in AD and PD underline the importance of regional iron homeostasis and cell-type context in modulating ferroptosis across different neurodegenerative diseases.

### 7.2. Ferroptosis Signatures in Alzheimer’s and Parkinson’s Disease

Disruption of iron metabolism and increased susceptibility to ferroptotic pathways are common features of AD and PD. Neurons in the brains of AD and PD patients present multiple hallmarks of ferroptotic stress in affected regions, including increased lipid peroxidation products and disrupted expression of proteins that regulate iron homeostasis [95,96,167,168,169]. These changes are also observed in animal models of AD. For example, ferroportin was downregulated in the brains of AD patients and in APPswe/PS1dE9 mice [112,170]. Moreover, loss of ferroportin was associated with memory impairment in a second murine AD model [170].

While the molecular signatures are broadly similar in both diseases, the cellular context differs. In AD, iron dysregulation predominates in cortical and hippocampal regions, impacting both neurons and glia (astrocytes and microglia). In contrast, PD pathology centers on the SNpc, where dopaminergic neurons and surrounding glial cells are particularly susceptible. Regional differences in the expression of ferroptosis regulators, such as GPX4, system Xc^−^, or FSP1, and their heterogeneous expressions in different cell types likely underlie these distinct vulnerabilities.

Recent advances in single-cell and spatial omics have enabled high-resolution dissection of ferroptotic mechanisms in neurodegeneration. In AD, Dang et al. (2022) [171] used single-cell RNA sequencing to dissect ferroptosis in astrocytes and identified FTH1 and SAT1 as key drivers of iron-induced lipid peroxidation, thereby linking ferroptosis to Aβ toxicity. Subsequent transcriptomic analyses revealed additional ferroptosis-related genes associated with hippocampal degeneration in AD [172,173].

In PD, single-nuclei RNA sequencing of a human-stem-cell-derived tri-culture system (microglia, neurons, and astrocytes) revealed that SEC24B is a regulator of iron-driven ferroptosis in microglia [165]. Zhang et al. (2024) [174] further linked α-synuclein aggregation to ferroptotic neuronal loss. Spatial transcriptomics was used to map transcriptional alterations in midbrain dopamine neurons, identifying molecular subtypes uniquely vulnerable to PD degeneration [175]. These spatial profiles confirmed the colocalization of α-synuclein aggregates with astrocyte clusters expressing low SLC7A11 and high SAT1 and ACSL4, implicating localized iron dyshomeostasis in disease progression [171].

Despite these insights, many studies rely on bulk transcriptomics and bioinformatics analyses, limiting cell-type-specific and spatial resolution. Furthermore, comparative analyses between AD and PD and across disease stages remain scarce. Emerging single-cell studies are beginning to fill these gaps. Shwab et al. (2024) [176] and Bhattarchaya et al. (2025) [177] investigated transcriptional changes in AD and PD brains at single-cell resolution. These studies replicated the roles of important AD or PD genes and pathways and identified shared alterations in cellular energy metabolism and stress response, mitochondrial ROS, inflammation, lipid signaling, protein folding, and protein degradation [176,177]. Notably, oligodendrocytes, the primary myelin-producing cells, emerged as uniquely involved in PD pathology, whereas AD pathology was more strongly associated with microglia and astrocytes [176,177,178].

Significantly, dysfunction of oligodendrocytes may be an early hallmark of neurodegeneration [176,177,179]. Oligodendrocytes are particularly vulnerable to neurodegenerative conditions because of their high metabolic demands and sensitivity to oxidative stress [180,181]. As oligodendrocytes are the primary iron-containing cells in the brain and play a crucial role in maintaining iron homeostasis for myelin synthesis through the expression of transferrin and ferritin [182], their degeneration can release excess iron into the local environment, potentially exacerbating oxidative damage and ferroptosis.

Overall, single-cell and spatial omics approaches have revealed that, while AD and PD share a common mechanism—excess iron-driven oxidative damage and ferroptosis—their cellular and regional contexts differ markedly, emphasizing the roles of microglia, astrocytes, and oligodendrocytes in ferroptosis. Future studies should therefore prioritize comparative, spatially resolved analyses across neurodegenerative diseases and disease stages to clarify temporal dynamics and pinpoint therapeutic targets.

### 7.3. Iron, Protein Aggregation, and Liquid-Liquid Phase Separation (LLPS)

AD is characterized by the accumulation of Aβ plaques and neurofibrillary tangles composed of hyperphosphorylated tau protein [183]. Protein aggregates in AD may act as centers for iron accumulation and ROS production, potentially creating local environments conducive to ferroptotic cell death.

Studies have shown that Aβ plaques serve as sites of iron accumulation, with metal ions becoming entrapped within the fibrillar structure of these protein aggregates [184]. Metals, in turn, may further promote Aβ aggregation by inducing conformational changes that enhance oligomerization and fibril formation [184,185,186]. Similar bidirectional relationships exist between iron and tau pathology, with iron promoting tau hyperphosphorylation and aggregation [116,187], whereas hyperphosphorylated tau disrupts normal axonal transport mechanisms, potentially including those responsible for iron trafficking [116].

In PD, the relationship between iron and disease pathology is bidirectional [188]. Iron dysregulation in PD is closely linked to the aggregation of α-synuclein, the primary component of Lewy bodies [132]. Iron directly influences α-synuclein aggregation by promoting protein oligomerization and fibril formation REF. Additionally, the *SNCA* mRNA has an IRE in its 5′ untranslated region that controls its translation, suggesting that iron also regulates α-synuclein levels [189]. Conversely, α-synuclein aggregates can bind iron and act as a local source of oxidative stress through iron-catalyzed reactions [190]. As in AD, this bidirectional relationship may create a self-reinforcing cycle in which iron promotes α-synuclein aggregation, and the resulting aggregates further disrupt iron homeostasis.

Recent research has highlighted the role of LLPS, a mechanism that controls biological and biochemical processes [191], in protein aggregation in neurodegenerative diseases. Many disease-associated proteins, including tau [192,193,194,195], Aβ [196,197,198], and α-synuclein [132,199,200,201,202,203], can undergo LLPS to form liquid droplets, which may represent early stages in the pathway leading to pathological aggregation [132,192,193,194,195,199,200,201,202,203]. The liquid condensates can mature over time into more solid-like structures, eventually leading to the formation of toxic aggregates and fibrils [204].

Iron appears to modulate this process, with iron binding potentially accelerating the transition from liquid droplets to more stable fibrillar aggregates [192,205,206]. Early reports connected LLPS-driven protein aggregation with ferroptotic stress, implying that iron not only promotes lipid peroxidation but also modulates how vulnerable proteins transition from liquid-like droplets to insoluble deposits [207].

The interplay between iron, protein aggregation, and LLPS creates a complex pathological landscape in which these processes reinforce each other, potentially accelerating disease progression. Targeting this interplay represents a promising therapeutic approach for interrupting the neurodegenerative cascade.

### 7.4. Mitochondrial ROS

The brain may also be uniquely vulnerable to ferroptosis in part because of its mitochondria-rich neurons and high energy demands [56]. The mitochondrial iron pool, which is normally tightly regulated for heme synthesis and iron-sulfur cluster assembly, becomes dysregulated under conditions of iron overload [208,209,210], increasing the formation of hydroxyl radicals via the Fenton reaction and leading to oxidative damage to mitochondrial DNA, proteins, and lipids [56,211].

A self-perpetuating cycle of ROS production and oxidative stress is particularly evident in AD and PD, where mitochondrial dysfunction, characterized by deficits in respiratory chain complexes, altered mitochondrial morphology, and impaired mitophagy, is well documented [56,212]. These early changes in disease progression heighten neuronal susceptibility to ferroptosis by increasing ROS production and compromising energy metabolism. In turn, iron overload and lipid peroxidation exacerbate mitochondrial damage, while dysfunctional mitochondria release additional ROS and labile iron, collectively fueling ferroptotic processes.

### 7.5. Neuroinflammation and Ferroptosis

Neuroinflammation is another well-established feature of both AD and PD, and accumulating evidence suggests that it contributes to disease progression partly through effects on iron homeostasis and ferroptosis sensitivity [95,213]. Neuroinflammation is triggered by the activation of microglia and astrocytes, which release proinflammatory factors that exacerbate neuronal damage [214]. Proinflammatory cytokines such as IL-1β, TNF-α, and IL-6 can alter the expression of iron transporters and storage proteins, leading to increased cellular iron uptake and decreased iron export [214] through the upregulation of TfR1 and DMT1 expression and the downregulation of ferroportin expression [214,215]. Iron overload, in turn, contributes to the labile iron pool, which, in the presence of ROS, catalyzes the Fenton reaction and exacerbates lipid peroxidation.

Microglia are professional phagocytes of the central nervous system and are essential for removing a range of debris, including apoptotic neurons, myelin fragments, oxidized lipids, and degenerating synapses. Significantly, their phagocytic capacity is modulated by AD risk genes and protective variants, including APOE4 and APOE3 [216,217,218,219,220,221]. RNA sequencing analysis further revealed a possible link between lipid metabolism, ferroptosis, and phagocytosis in microglia [165]. Activated microglia can also release iron from their stores, increasing extracellular iron levels in the microenvironment surrounding neurons [222]. This iron can be taken up by neurons, potentially overwhelming their iron regulatory mechanisms and promoting ferroptotic cell death.

Inflammatory cascades typically involve reciprocal signaling among glial cells. The release of damage-associated molecular patterns from ferroptotic neurons [223] can further activate microglia and astrocytes, creating a feedforward cycle of inflammation and cell death. Oligodendrocytes can also modulate astrocyte and microglial activity via secreted factors; conversely, astrocytes and microglia produce signals that influence oligodendrocyte maturation and survival [165,213,224]. This intercellular cross-talk can perpetuate neuroinflammation.

### 7.6. Aging and Ferroptosis Susceptibility

As individuals age, different iron complexes tend to accumulate in brain regions associated with motor and cognitive decline [96,225]. The disruption of normal iron homeostasis may contribute to the altered cellular iron distribution and subsequent regional accumulation observed in neurodegenerative disorders.

The vulnerability of neurons to ferroptosis can also be exacerbated by age-related decreases in antioxidant defenses. For example, aging-related changes in iron handling and antioxidant defenses may contribute to the age-dependent increase in AD risk. AD and PD neurons exhibit decreased activity of the GPX4 system, which normally protects against ferroptosis by converting lipid hydroperoxides into nontoxic compounds [167,168].

This phenomenon may be particularly relevant in PD, as the high content of polyunsaturated fatty acids in neuronal membranes, combined with the oxidative environment created by dopamine metabolism and iron accumulation, makes these cells especially susceptible to ferroptotic damage [226,227,228,229]. In addition, age-related disruption of BBB function may lead to increased iron accumulation in the brain, further enhancing neuronal vulnerability to ferroptosis [230,231].

## 8. Iron Dyshomeostasis and Ferroptosis: Implications for Other Neurodegenerative Conditions

While AD and PD are the two primary examples of neurodegenerative diseases linked to iron overload and ferroptosis, converging evidence increasingly establishes ferroptosis and iron dyshomeostasis as critical pathogenic mechanisms underlying other neurodegenerative conditions, including ALS, HD, and MS. Each disease exhibits unique features—demyelination, motor neuron loss, or striatal atrophy—but may share this common mechanism of iron-induced oxidative cell death, warranting further investigation.

### 8.1. Amyotrophic Lateral Sclerosis (ALS)—Ferroptotic Motor Neuron Loss

ALS is characterized by the selective degeneration of motor neurons in the cortex, brainstem, and spinal cord [232]. Neuroimaging and histological studies have provided evidence of significant iron accumulation in affected regions in both patients and animal models, suggesting a role for ferroptosis in the distinctive pattern of motor neuron loss in ALS. For example, MRI has revealed abnormal hypointense signals in the primary motor cortex of ALS patients, which pathological analysis has attributed to iron-loaded microglia in the deep cortical layers [233]. This iron deposition in the motor cortex correlated with upper motor neuron impairment and was apparent even in early (premanifest) stages of disease [233]. Similarly, postmortem analyses and susceptibility-weighted imaging have revealed excessive iron in the spinal cord and various subcortical nuclei of ALS patients [234]. In transgenic ALS mouse models (e.g., SOD1 mutations), ventral spinal motor neurons have shown progressive iron accumulation correlating with disease progression [234]. These patterns indicate that both neurons and glia in ALS patients show enhanced iron loading in regions undergoing neurodegeneration.

At the molecular level, recent findings strongly link ferroptosis to ALS pathogenesis. In spinal cord tissue from both sporadic and familial ALS patients, researchers have reported a marked depletion of GPX4 [235], indicating an impaired ability to detoxify lipid peroxides. Notably, the same signature is recapitulated in multiple ALS mouse models, where GPX4 loss in the spinal cord occurs early and universally during disease development [235]. The loss of this ferroptosis suppressor, together with the dysregulation of other antioxidant pathways, such as NRF2 signaling and glutathione synthesis, creates high susceptibility to iron-mediated lipid peroxidation in ALS neurons [235]. Indeed, lipid peroxidation damage, which is consistent with ferroptotic injury, is a prominent feature of degenerating motor neurons in individuals with ALS [236]. Conversely, genetic or pharmacological enhancement of GPX4 activity has been shown to delay the onset of paralysis, preserve motor neurons, and prolong survival in ALS models (SOD1G93A) [237]. This neuroprotection was accompanied by an attenuation of lipid peroxidation in the spinal cord.

In addition to neurons, glial cells in ALS also contribute to ferroptosis-related pathology. Iron import and storage proteins (DMT1, transferrin receptor, ferritin) are abnormally upregulated in ALS models, indicating that microglia and astrocytes may sequester excess iron and propagate oxidative stress [234]. Therefore, iron accumulation may amplify neuroinflammation, further contributing to motor neuron vulnerability via ferroptotic mechanisms. Indeed, a ferroptosis signature identified in a recent patient study has been shown to act as a prognostic biomarker panel for ALS [238]. Together, these data support a model in which iron overload and impaired antioxidant defenses trigger ferroptosis of motor neurons in ALS. The characteristic motor neuron loss in ALS patients, both in the cortex and the spinal cord, may therefore stem from iron-induced lipid peroxidation.

### 8.2. Huntington’s Disease (HD)—Iron, Mitochondria, and Striatal Neurodegeneration

HD is an inherited neurodegenerative disorder marked by progressive loss of medium spiny neurons in the striatum (caudate and putamen), leading to motor dysfunction (chorea) and cognitive decline [239]. Emerging evidence implicates iron dyshomeostasis and ferroptosis in the distinctive striatal pathology of HD. Compared with healthy controls, brain imaging studies have consistently revealed elevated iron content in the basal ganglia, particularly in the caudate, putamen, and globus pallidus, of both premanifest and symptomatic HD individuals [240]. This accumulation of iron in the striatum increases with disease severity and duration, suggesting that it is both an early event and a continuing process throughout HD progression [234]. Postmortem analyses have confirmed increased ferric iron in the striatal neurons of HD patients [240].

Mechanistically, the mutant huntingtin protein appears to disrupt cellular iron handling by increasing the expression of iron importers and promoting iron accumulation in neuronal mitochondria [241]. This mitochondrial iron overload contributes to lipid ROS generation, potentially linking iron directly to the energy deficits and oxidative damage characteristic of HD neurons [241,242]. Both HD mouse models and patient tissues exhibit increased lipid peroxidation and oxidative damage in striatal cells, which is correlated with the degree of neurodegeneration and severity of motor symptoms [242,243,244,245]. The accumulation of toxic lipid peroxidation byproducts in the corpus striatum has been associated with worsened motor performance [246,247], suggesting that ferroptosis is a plausible mechanism for the selective vulnerability of the striatum in HD.

Transcriptomic studies further support this connection, revealing the dysregulation of multiple ferroptosis regulators in HD patient brains. These include altered expression of antioxidant enzymes and iron transport proteins and activation of lipid-peroxide-producing enzymes such as 5-lipoxygenase (ALOX5) in both neurons and microglia of the striatum [242,248]. These changes suggest that neurons and glial cells in HD may engage in ferroptotic pathways that contribute to the overall neurodegenerative process. In summary, HD pathology features regional iron accumulation that triggers mitochondrial dysfunction and oxidative stress, ultimately leading to ferroptosis of vulnerable striatal neurons.

### 8.3. Multiple Sclerosis (MS)—Iron, Ferroptosis, and Demyelination

MS is an inflammatory demyelinating disease [249] and converging evidence suggests that iron dysregulation contributes significantly to its pathology. Abnormal iron accumulation is observed in so-called “iron rim” lesions, where iron-laden microglia and oligodendrocytes contribute to chronic tissue damage [250,251]. Postmortem and magnetic resonance imaging (MRI) studies of progressive MS have revealed chronic active plaques with iron-laden microglia at the edges that are more destructive, expand over time, and are characterized by a ferroptosis signature [252,253,254]. This regional iron overload may accelerate oxidative injury and impair remyelination processes.

The cellular mechanisms connecting iron and MS pathology are becoming increasingly clear. Oligodendrocytes, which are essential for myelin production, are naturally enriched in iron for myelin synthesis, which may render them particularly susceptible to ferroptosis under conditions of oxidative stress [255]. Excess iron in these cells promotes oxidative stress and lipid peroxidation, creating ideal conditions for ferroptotic cell death. Single-cell RNA sequencing and animal studies have confirmed that oligodendrocyte death in MS is accompanied by molecular hallmarks of ferroptosis [253,256]. At the molecular level, experimental autoimmune encephalomyelitis (EAE) mouse models have demonstrated increased expression of iron import proteins (e.g., TfR1) and markers of ferritinophagy (NCOA4), which are correlated with oligodendrocyte death. Pathological iron accumulation appears to result from altered expression and function of iron transport proteins at the blood-brain barrier, exacerbated by inflammatory cytokines and oxidative stress that modulate key iron transporters such as transferrin receptor, divalent metal transporter 1, and ferroportin. These changes lead to increased iron influx or impaired efflux, promoting tissue iron accumulation.

Importantly, therapeutic approaches targeting ferroptosis have shown promise in MS models. Treatment of EAE mice with small-molecule ferroptosis inhibitors (liproxstatin-1 or ferrostatin-1) significantly alleviates clinical severity and demyelination [253,257]. Consistent with ferroptotic mechanisms, neurons in EAE patients exhibit reduced levels of the antiferroptotic enzyme glutathione peroxidase 4 (GPX4) and elevated lipid peroxidation, with GPX4 depletion correlated with neuronal death [253,257]. An elegant study revealed that a histone methyltransferase (G9a) can drive MS progression by repressing neuronal GPX4 and other antiferroptosis genes, underscoring the disease relevance of ferroptotic pathways [253,258].

The ferroptosis-inflammation connection appears to be bidirectional in MS. As neurons undergo ferroptosis, they can secrete factors that further activate infiltrating T cells (Th1/Th17), exacerbating autoimmune inflammation [253,259]. This creates a vicious cycle linking iron overload directly to the demyelination and neuroinflammation that characterize MS pathology. These findings collectively suggest that iron-mediated ferroptosis in oligodendrocytes and neurons may be a fundamental mechanism underlying MS progression.

### 8.4. Converging Mechanisms: Iron-Mediated Ferroptosis as a Common Pathway in Neurodegeneration

The evidence presented for ALS, HD, and MS reveals striking parallels with the iron dysregulation previously discussed in AD and PD, suggesting that iron-mediated ferroptosis represents a unifying mechanism in neurodegeneration. Despite their distinct clinical presentations and affected neural populations, these conditions share several key features: region-specific iron accumulation, impaired antioxidant defenses, and vulnerability to lipid peroxidation. In each disease, iron dysregulation occurs in precisely the most affected regions, motor neurons in ALS patients, striatal neurons in HD patients, and oligodendrocytes in MS patients, suggesting that iron-catalyzed oxidative damage may determine regional vulnerability patterns. Moreover, the molecular signatures of ferroptosis, particularly GPX4 depletion and lipid peroxidation, appear consistently across these disorders, often preceding overt neurodegeneration.

## 9. Clinical Translation: Iron Chelation and Overcoming the Blood-Brain Barrier

Given the growing evidence implicating ferroptosis in neurodegeneration, targeting this cell death pathway therapeutically offers a promising approach for treating different neurodegenerative diseases. Translating knowledge of iron-driven pathology into patient therapies has focused largely on iron chelators, which bind and sequester excess iron before it can participate in damaging Fenton reactions [260,261].

Iron chelation has been explored as a disease-modifying strategy in Parkinson’s disease due to the excess iron found in the basal ganglia of PD patients [262]. Among these, deferiprone is already approved for treating systemic iron overload in β-thalassemia patients [263]. Deferiprone, a brain-penetrant iron chelator, has recently undergone clinical trials involving early PD patients with mixed results. A recent meta-analysis of randomized trials concluded that deferiprone did not significantly improve motor function (based on Unified Parkinson’s Disease Rating Scale scores) relative to placebo [262], even though significant reductions in iron concentration were achieved in the substantia nigra and other deep brain nuclei [262]. In line with these findings, a 2022 multicenter trial in early PD reported no slowing of disease progression with an 18-month course of deferiprone, despite evidence of iron depletion in target regions [262,264,265]. Thus, to date, iron chelation in PD has shown target engagement without clear clinical benefit, and expert consensus is that current evidence does not support its routine use as a neuroprotective therapy [262]. Ongoing studies are examining whether specific subpopulations of PD patients or different dosing regimens might yet reveal a therapeutic benefit.

Few clinical trials have investigated iron chelation therapy for AD thus far, partly because AD pathology is more diffuse, and the direct correlation between iron deposition and clinical symptoms is less straightforward. A recent randomized trial of deferiprone in patients with amyloid-confirmed early AD produced a counterintuitive outcome: patients on deferiprone deteriorated more rapidly than those on placebo [266,267]. Over 12 months, deferiprone-treated individuals showed markedly accelerated cognitive decline, even though quantitative MRI confirmed significant iron reduction in the hippocampus [266,267]. These findings imply that indiscriminate chelation of brain iron may either disrupt essential iron-dependent processes or exacerbate neuronal vulnerability in AD. One interpretation is that brain iron elevation in AD could be a reactive, possibly protective sequestration mechanism (for instance, immobilizing iron in plaques or ferritin); thus, aggressive iron removal might inadvertently release redox-active iron or deprive neurons of necessary iron [266,267]. Alternatively, the dose and duration of chelation might have been inappropriate, leading to functional iron deficiency in the brain [266,267]. Whatever the underlying reason, the stark contrast with the PD trials highlights that the efficacy and safety of iron chelation differ between neurodegenerative diseases, warranting cautious, disease-specific evaluation.

The mixed clinical outcomes of broad-spectrum iron chelators have prompted the development of more selective iron-targeting strategies. One example is the BBB-permeable compound ATH434, an iron chaperone, developed by Alterity Therapeutics, which binds and neutralizes excess labile iron without depleting physiological iron stores. Notably, ATH434 has a much lower affinity for ferric iron than do traditional chelators and binds iron in a reversible manner, meaning it will not scavenge iron essential for normal cells [268]. In contrast to standard chelators (which can trigger the Fenton reaction by oxidizing Fe^2+^ and inadvertently generating ROS), ATH434 causes minimal oxidative by-product formation [268]. These properties allow it to selectively target pathogenic iron pools while preserving normal iron-dependent processes. Such next-generation agents are now in clinical trials for synucleinopathies (multiple system atrophy and PD), reflecting a promising direction for iron-targeted therapy.

In addition to chelation, enhancing neuronal antioxidant defenses, such as GPX4 activity [269,270,271,272,273,274,275], or alternative pathways, such as FSP1-mediated ubiquinol regeneration, could suppress ferroptosis. Since reactive astrocytes, microglia, and oligodendrocytes appear to be early hallmarks of neurodegeneration and contribute to iron dysregulation and the inflammatory microenvironment, therapies aimed at normalizing their function could also be beneficial. Combination therapies that address both iron imbalance and oxidative stress (for example, the use of ferrostatin or mitochondrion-targeted antioxidants) may be especially promising [276,277,278]. Early intervention is also likely key, as iron accumulation and ferroptotic stress can precede overt clinical symptoms.

Targeting ferroptosis has also shown promise in preclinical models of MS, ALS, and HD, suggesting that modulating this cell death pathway could be a viable therapeutic strategy to slow or mitigate these otherwise intractable neurodegenerative diseases. The cell-specific vulnerabilities of motor neurons in ALS, medium spiny neurons in HD, and oligodendrocytes in MS may reflect different cellular capacities for managing iron and combating lipid peroxidation. Understanding these differences within the common framework of iron-mediated ferroptosis will likely be necessary to guide the development of disease-specific therapeutic approaches. In fact, new agents like ATH434 discussed above exemplify a shift toward precision iron modulation. Going forward, tailoring iron-based therapies to each disease’s specific iron dysregulation profile and combining iron modulation with other neuroprotective strategies will likely be crucial for therapeutic success.

A key hurdle in any therapeutic approach targeting ferroptosis in the brain is ensuring that the drug reaches its neuronal targets. New drug delivery strategies, such as encapsulating chelators or ferroptosis inhibitors in nanoscale carriers coated with BBB-penetrating ligands, are being pursued [279,280]. Nanocarriers constructed from lipids, polymers, or biocompatible metals can release their cargo in a controlled manner within the brain, potentially increasing both the efficacy and safety profiles. Another emerging approach is to modify small-molecule inhibitors, either chemically or by linking them to shuttle peptides so that they exploit endogenous transport systems across the BBB [281].

The trial outcomes observed with deferiprone in PD and AD underscore both the promise and the complexity of ferroptosis-focused therapies. As research advances, the refinement of ferroptosis-targeting strategies, novel BBB-penetrating platforms, and the identification of biomarkers that identify patients who stand to benefit most may thus tip the balance toward successful clinical translation.

## 10. Challenges and Future Directions

Despite these advances, many open questions and knowledge gaps remain. While an increasing number of studies support an association between iron overload, ferroptosis, and neurodegeneration, this association is not causal. The precise temporal sequence linking iron accumulation to protein aggregation is still under investigation, and reliable biomarkers of ferroptosis in humans are still lacking. Future research integrating single-cell omics, longitudinal neuroimaging, and clinical trials will be crucial for refining our understanding of a causative link and the underlying molecular mechanisms. Such studies will also be necessary for devising therapeutic strategies and for identifying the patients most likely to benefit from ferroptosis-targeting interventions. Moreover, an emerging area of interest is the link between the gut microbiome, ferroptosis, and neurodegenerative diseases [282,283,284].

Many challenges also remain in the development of more effective ferroptosis-targeted therapeutics [270]. The complexity of neurodegenerative diseases, involving multiple cell types and pathological processes, necessitates careful consideration of timing and regional targeting in therapeutic approaches.

Finally, we note that ferroptosis is one of several regulated cell death pathways implicated in neurodegenerative diseases, which often feature a convergence of multiple cell death mechanisms [285,286]. Common pathogenic factors, such as stress from misfolded proteins, mitochondrial dysfunction, and chronic neuroinflammation, can initiate a spectrum of death processes including apoptosis, necroptosis, pyroptosis, and ferroptosis [285,286,287]. For instance, in AD brains, alongside signs of ferroptotic lipid peroxidation, researchers have observed evidence of apoptotic cell death and inflammatory pyroptosis in degenerating neuronal populations [285]. Similarly, in PD, dopaminergic neurons exhibit not only ferroptosis-associated iron overload but also apoptotic markers, suggesting overlapping modes of cell death [286]. Thus, future mechanistic studies should focus on the interplay of different cell death pathways. Likewise, therapeutic interventions may need to address multiple death pathways to effectively protect neurons.

## 11. Conclusions

Ferroptosis represents a compelling molecular pathway that unites iron dyshomeostasis and oxidative stress with neuronal loss in neurodegenerative diseases. The central nervous system’s high metabolic demands, lipid-rich membranes, and reliance on tight iron regulation set the stage for iron-driven damage when homeostasis falters. Mounting data suggest that pathologic iron accumulation not only correlates with hallmark protein aggregates but also actively drives disease progression via ferroptotic death.

Continued efforts to elucidate ferroptosis mechanisms in neurons, refine iron-targeting therapies, and improve drug delivery across the BBB hold promise for more effective disease-modifying treatments. By focusing on iron homeostasis in the brain and linking it directly to ferroptosis, we gain a more cohesive and translational perspective on the pathophysiology of neurodegenerative disorders and on potential strategies to halt or slow these diseases.

## Data Availability

No new data were created.
